# Interplay between resting heart rate variability, daily affective dynamics and mental health difficulties in autistic youths

**DOI:** 10.1038/s41598-025-34614-y

**Published:** 2026-01-06

**Authors:** Laura Ilen, Julie Husmann, Clémence Feller, Maude Schneider

**Affiliations:** Clinical Psychology Unit for Intellectual and Developmental Disabilities, Faculty of Psychology and Educational Sciences, University of Geneva, 40, Boulevard du Pont-d’Arve, 1205 Geneva, Switzerland

**Keywords:** Diseases, Health care, Neuroscience, Psychology, Psychology

## Abstract

Repeated exposure to external stressors could lead to altered autonomic functioning, contributing to difficulties in emotion regulation and the development of mental health difficulties. This pathway is important to investigate in autistic youths who are vulnerable to develop stress-related mental health difficulties. We investigated the interplay between resting heart rate variability (HRV), fluctuations of daily affects over time (daily affective dynamics) and mental health symptoms (internalizing, externalizing and social anxiety symptoms) in 27 autistic (M_age_=17.66) and 24 non-autistic (M_age_=19.86) adolescents and young adults. Resting HRV was collected using a mobile electrocardiogram. Ecological Momentary Assessment assessed daily affective dynamics (affective instability and affective reactivity to stress). Mental health symptoms were assessed with questionnaires. Resting HRV did not differ significantly between the groups. HRV was not directly associated with mental health symptoms in analyses corrected for multiple comparisons, but affective reactivity to stress mediated the association between HRV and internalizing symptoms. Finally, differences emerged in the relationship between HRV and daily affective dynamics between autistic and non-autistic individuals. Our results suggest that increased stress reactivity may be a mechanism linking altered autonomic functioning to mental health difficulties, offering important insights for targeting clinical interventions.

**Supplementary Information:**

The online version contains supplementary material available at 10.1038/s41598-025-34614-y.

## Introduction

The existence of a pathway between exposure to environmental stressors and subsequent mental health difficulties has been consistently demonstrated in the literature and may be mediated by altered functioning of the body’s physiological stress response systems. Indeed, both major stressful life events and minor daily hassles (which are perceived as stressful), modify the individual’s physiological and behavioral responses^1^. The physiological stress response is characterized by increased heart rate, which is modulated by the autonomic nervous system (ANS) through the sympathetic and parasympathetic branches^2^. Heart rate variability (HRV)—the variability in time intervals between consecutive heart beats—is considered as an indicator of the interplay between sympathetic and parasympathetic activity^3^ and an important marker of the ability to adapt to environmental changes^4^. A reduction in resting HRV may arise from repeated/chronic stress exposure, which could lead to chronic overactivation of physiological systems, resulting in long-lasting alterations in stress responses and increased vulnerability to negative health outcomes^1^. Reduced resting HRV has been observed in various psychiatric conditions^5^ and has been associated with the severity of psychiatric symptoms in non-clinical populations^6^, hence it is considered a transdiagnostic biomarker in psychiatry^7^.

A possible underlying mechanism linking stress exposure, HRV and mental health difficulties may be the capacity of emotion regulation. Altered development of the ANS (e.g. due to early stress exposure) might contribute to difficulties in adaptive emotion regulation^8^, which is observed across psychiatric conditions^9,10^. More specifically, reduced resting HRV is seen as a biomarker of nonadaptive emotion regulation, whereas higher HRV has been associated with more adaptive regulation of emotions^11^. As individuals face a variety of minor stressors on a daily basis, the ability to regulate affects and its link with HRV appears important to investigate in daily life. The Ecological Momentary Assessment (EMA), a structured diary method that enables repeated assessments in the flow of daily life^12^ is a suitable technique for investigating how affects fluctuate over time in individuals’ daily lives (i.e., daily affective dynamics) in relation to stressors and emotion regulation. Aspects of daily affective dynamics include *affective instability*, i.e., large and frequent mood shifts over time^13^, and *affective reactivity to stress*, i.e., increase in negative affects in relation to perceived daily stressors^14^. While affective reactivity to stress models the change in affect as a function of context (stress), affective instability reflects the within-individual changes in affect between different moments. Affective instability is therefore thought to reflect both one’s sensitivity to the environment and one’s emotion regulation strategies^15^ (for example, rumination could increase negative affects in absence of acute stressors). Higher affective instability and increased affective reactivity to stress thus both reflect nonadaptive regulation of daily affects. They have been associated with lower resting HRV^16,17^ and have shown to be increased in various clinical populations^14,18^. However, to date, studies have investigated the interplay between HRV and daily affective dynamics in the general population, although this relationship may be particularly relevant for populations with an increased vulnerability to stress and mental health difficulties during development.

One such population includes individuals with autism spectrum disorder (ASD), a neurodevelopmental condition whose core characteristics (social difficulties, restricted/repetitive behaviours and sensitivity to sensory information) might increase individuals’ vulnerability to external and internal stressors^19,20^, possibly altering the adaptive modulation of physiological responses. Although several previous studies point in this direction, showing reduced resting HRV in autistic individuals^21^, the results are globally mixed and are thought to be influenced by inter-individual factors^22^, such as the presence of co-occurring mental health conditions^23^ or medication status^24^. Some associations have previously been demonstrated between altered autonomic functioning and the severity of anxiety symptoms in autism^23,25^, although studies on the topic remain scarce. Furthermore, as emotion regulation difficulties are reported in autism^26^, one might hypothesize that individuals’ ability to regulate daily affects could contribute to the inter-individual variability in HRV. Previous research has shown associations between nonadaptive regulation of daily affects (i.e., greater affective instability and heightened affective reactivity to stress) and the severity of mental health symptoms in autism^27,28^, raising the question of the possible role of altered autonomic functioning. However, no studies have investigated the relationship between autonomic functioning (HRV), regulation of daily affects and mental health symptoms in autistic individuals.

**Fig. 1:**
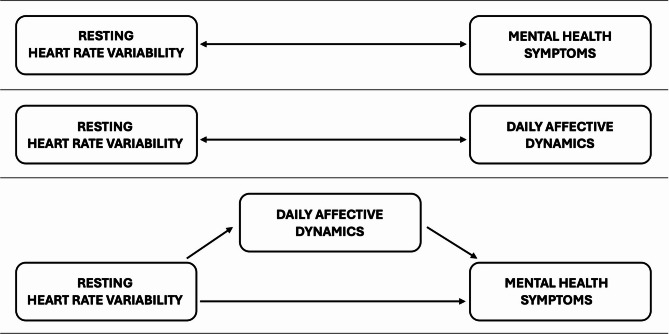
Diagram of the study aims and hypotheses.

In the current study, we aimed to investigate whether resting HRV would be cross-sectionally associated with mental health symptoms through daily affective dynamics in autistic and non-autistic youths. Based on previous literature, particularly a recent review^22^, we expected to observe high inter-individual variability in HRV among all individuals and no alterations in HRV at the group level among autistic youths. We therefore decided to conduct the analyses by combining all participants (unless significant group differences in HRV would be observed), in order to investigate associations transdiagnostically in autistic and non-autistic youths and increase the statistical power. First, we hypothesized to observe an association between low resting HRV and more severe mental health symptoms (internalizing, externalizing and social anxiety symptoms). Social anxiety was investigated alongside internalizing and externalizing symptoms because it provides an additional measure not accounted for in these dimensions, represents a common comorbidity in autism^29^ and has been associated with reduced HRV^30^. Second, we expected lower resting HRV to be associated with greater instability of positive (PA) and negative (NA) affects. Third, we hypothesized that resting HRV would moderate the association between daily-life stress and NA. Finally, we hypothesized that affective instability and affective reactivity to stress would mediate the association between resting HRV and mental health symptoms. Study aims and hypotheses are presented in Fig. 1.

## Results

### Sample characteristics

Sample characteristics are presented in Table 1. The final sample consisted of 27 autistic participants and 24 non-autistic participants. Age and body mass index (BMI) were higher in the non-autistic group compared to the autistic group. Sex and intelligence quotient (IQ) did not significantly differ between the groups.

**Table 1:** Demographic characteristics and variables of interest.

	Non-autistic (*n* = 24)	Autistic (*n* = 27)	Test statistic	*p*-value
Age in years, mean (S.D.)	19.86 (3.55)	17.66 (4.08)	*U* = 451	0.016*
Sex (*n* M/F)	11/13	10/17	*X*^*2*^(1) = 0.41	0.524
Body Mass Index (BMI), mean (S.D.)	23.63 (3.71)	21.83 (7.50)	*U* = 460	0.004**
Intelligence Quotient (IQ), mean (S.D.)	113.75 (13.15)	110.74 (14.25)	*U* = 352.5	0.597
Regular smoking of tobacco, *n* (%)	1 (4.2%)	4 (14.8%)		
rMSSD, mean (S.D.)	41.65 (19.95)	43.97 (30.12)	*U* = 336	0.830
HF, mean (S.D.)	312.67 (236.90)	470.11 (655.62)	*U* = 320	0.948
EMA, mean (S.D.)				
Completed beeps	32.96 (6.69)	30.37 (9.29)	*U* = 379	0.303
% Completed beeps	68.7%	63.3%		
Response time (s)	146.68 (92.11)	193.24 (114.24)	*T =* 2.40	0.02*
EMA Period, *n* (structured/no structured activity)	12/12	5/22	*X*^2^(1) = 5.67	0.017*
PA instability	0.92 (0.51)	1.25 (1.06)	*U* = 297	0.620
NA instability	0.4 (0.41)	1.14 (1.06)	*U* = 155	0.001**
Event stress reactivity (b)	0.08 (0.06)	0.16 (0.10)	*U* = 152	< 0.001***
Activity stress reactivity (b)	0.14 (0.06)	0.18 (0.08)	*U* = 225	0.063
Social stress reactivity (b)	0.28 (0.08)	0.35 (0.14)	*U* = 249	0.161
ABCL/CBCL, mean (S.D.)				
Externalizing symptoms	45.46 (8.30)	56.81 (6.49)	*U* = 82	< 0.001***
Internalizing symptoms	47.00 (9.19)	75.56 (8.40)	*U* = 0	< 0.001***
ASR/YSR, mean (S.D.)				
Externalizing symptoms	51.00 (8.43)	59.00 (7.09)	*U* = 146.5	< 0.001***
Internalizing symptoms	54.04 (9.26)	72.85 (8.19)	*U* = 42	< 0.001***
SIAS, mean (S.D.)	26.33 (14.82)	54.22 (13.73)	*U* = 58.5	< 0.001***
Psychiatric diagnosis, *n* (%)				
Anxiety disorder		20 (74.1%)		
Mood disorder		4 (14.8%)		
Post-traumatic stress disorder		2 (7.4%)		
Other^a^		5 (18.5%)		
Other neurodevelopmental disorder, *n* (%)				
ADHD		11 (40.7%)		
Psychotropic medication, *n* (%)				
Antidepressants		11 (40.7%)		
Antipsychotics		5 (18.5%)		
Anxiolytics		5 (18.5%)		
Psychostimulants		6 (22.2%)		

The same participant can have > 1 diagnoses and medication.

HF, High-frequency power; rMSSD, Square root of the mean squared differences of successive R–R intervals; EMA, Ecological Momentary Assessment, PA, Positive Affects; NA, Negative Affects; ABCL/CBCL, Adult/Child Behavior Checklist; ASR/YSR, Adult/Youth Self Report; SIAS, Social Interaction Anxiety Scale.

*p < 0.05; **p < 0.01; ***p < 0.001.

^a^Other diagnoses include obsessive-compulsive disorder (*n* = 1), insomnia disorder (*n* = 1), oppositional defiant disorder (*n* = 1) and bulimia (*n* = 2).

Regarding EMA measures, the compliance did not differ between autistic (817 valid responses) and non-autistic participants (778 valid responses), but the autistic group took longer to respond to notifications. Moreover, the period during which participants took part in the EMA protocol differed between the groups: autistic participants participated less often during school/work and more often during a period without structured activity in comparison to non-autistic participants.

### Group comparisons for resting HRV

**Fig. 2:**
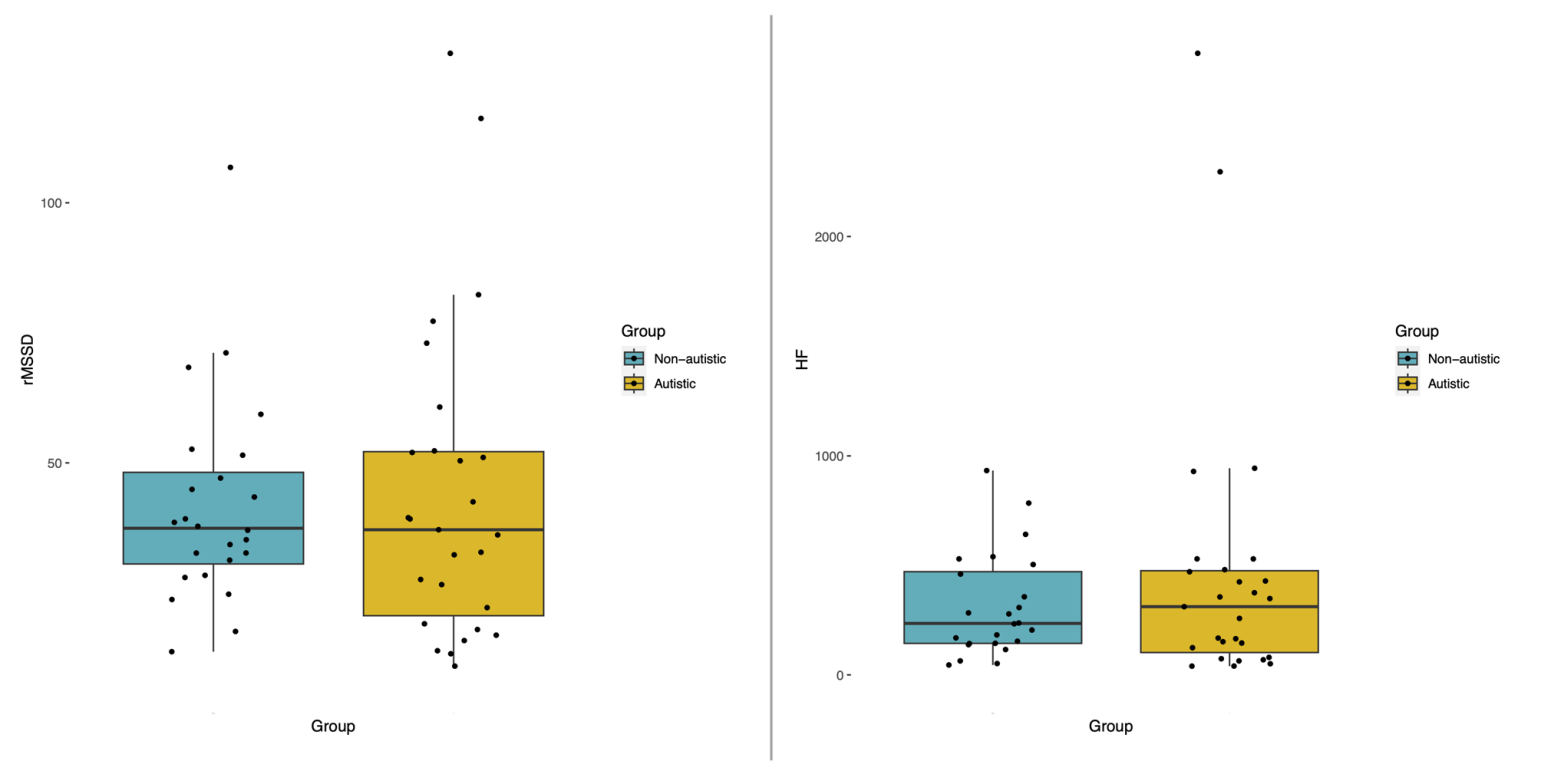
Distribution of HRV parameters (rMSSD and HF-HRV) in each group.

Multiple linear regression was performed to investigate group differences in HRV parameters: rMSSD (the square root of the mean squared differences of successive R–R intervals) and HF (high frequency power). For rMSSD, no significant group differences were observed (*b *= − 0.06, 95% CI [− 0.21,0.07], *t*(48) = − 0.89, *p* = 0.375). The model explained a weak proportion of the variance (R^2^ = 0.06, F(2, 48) = 1.60, *p* = 0.211, adj. R^2^ = 0.02). No significant group differences were observed for HF either (*b *= − 0.05, 95% CI [− 0.30,0.19], *t*(48) = − 0.40 *p* = 0.669), with the model again explaining a weak proportion of the variance (R^2^ = 0.07, F(2, 48) = 1.84, *p* = 0.171, adj. R^2^ = 0.03). Therefore, all the participants were combined for further analyses. However, higher variability in the two HRV parameters was visually observed within autistic individuals (Fig. 2). Of note, the HRV parameters were strongly correlated with each other (*rho* = 0.91, *S* = 1988.00, *p* < 0.001).

### Resting HRV and mental health

Detailed results of the multiple regression models on the effect of HRV on mental health symptoms are presented in Table 2. No significant associations were observed between HRV and mental health symptoms after correction for multiple comparisons.

**Table 2:** Results of the multiple regression models on the association between HRV parameters and mental health symptoms in the whole sample.

	β (95% CI)	t	*p*	*R*^2^ (adj. *R*^2^)	F(2, 48)	*p*
Social anxiety						
rMSSD	− 0.002 (− 0.006, 0.002)	− 1.12	0.267	0.07 (0.03)	1.85	0.169
HF	− 0.003 (− 0.009, 0.003)	− 0.96	0.341	0.09 (0.05)	2.25	0.117
Internalizing (self)						
rMSSD	− 0.006 (− 0.01, − 0.0009)	− 2.37	0.022	0.15 (0.11)	4.13	0.022
HF	− 0.008 (− 0.02, 0.002)	− 1.66	0.104	0.12 (0.08)	3.22	0.049
Externalizing (self)						
rMSSD	− 0.004 (− 0.01, 0.004)	− 0.96	0.342	0.07 (0.03)	1.67	0.199
HF	− 0.002 (− 0.02, 0.01)	− 0.33	0.739	0.07 (0.03)	1.81	0.175
Internalizing (parent)						
rMSSD	− 0.004 (− 0.008, 0.0005)	− 1.76	0.085	0.01 (0.07)	2.81	0.07
HF	− 0.003 (− 0.01, 0.004)	− 0.83	0.408	0.08 (0.04)	2.12	0.131
Externalizing (parent)						
rMSSD	− 0.001 (− 0.009, 0.007)	− 0.3	0.765	0.05 (0.009)	1.23	0.301
HF	0.002 (− 0.01, 0.01)	0.25	0.801	0.07 (0.03)	1.78	0.179

*p*-values did not survive multiple comparison correction (B–H threshold: *p* = 0.005). Analyses conducted using multiple linear regression models, controlling for recent eating.

HF, High-frequency power; rMSSD, Square root of the mean squared differences of successive R–R intervals.

### Resting HRV and daily affective dynamics

When investigating the effect of HRV on daily affective dynamics in the whole sample, we observed that rMSSD and HF moderated the association between activity-related stress and NA, indicating that individuals with lower resting HRV showed increased affective reactivity to activity-related stress (Table 3). In contrast, HRV was not associated with affective reactivity to stress in other contexts or with affective instability.

**Table 3:** The effect of HRV parameters on affective dynamics in the whole sample.

	β (95% CI)		t	*p*
Outcome: PA instability^1^				
rMSSD	− 0.40 (− 0.98, 0.17)		− 1.42	0.162
HF	− 0.21 (− 0.54, 0.11)		− 1.32	0.194
Outcome: NA instability^1^				
rMSSD	− 0.06 (− 0.57, 0.45)		− 0.25	0.806
HF	− 0.36 (− 0.31, 0.27)		− 0.13	0.894
Outcome: NA^2^				
Event stress × rMSSD	− 0.11 (− 0.26, 0.05)			0.181
Event stress × HF	− 0.06 (− 0.15, 0.02)			0.158
Activity stress × rMSSD	− 0.20 (− 0.36, − 0.03)			0.022*
Activity stress × HF	− 0.12 (− 0.21, − 0.02)			0.016*
Social stress × rMSSD	− 0.01 (− 0.33 to 0.31)			0.953
Social stress × HF	0.04 (− 0.15, 0.23)			0.704

Recent eating was controlled for in all the models. The average level of PA/NA was controlled for in the analyses on affective instability.

HF, High-frequency power; NA, Negative affects; PA, Positive affects; rMSSD, Square root of the mean squared differences of successive R–R intervals.

*p < 0.05.

^1^Robust regression models.

^2^Multilevel regression models.

### Mediation analysis

Finally, a mediation analysis was conducted to investigate whether daily affective dynamics mediate the association between HRV and mental health symptoms. First, associations between daily affective dynamics and mental health symptoms were tested using linear regression in the whole sample and results are reported in supplementary material. After multiple comparison correction, NA instability and affective reactivity to event-related stress were associated with all the measures of symptoms, except for parent-reported externalizing symptoms. Affective reactivity to social stress was associated with self- and parent-reported internalizing symptoms as well as self-reported externalizing symptoms. Affective reactivity to activity-related stress was associated with self-reported internalizing symptoms. PA instability showed no significant associations with any symptoms.

Then, bootstrap mediation was performed using the variables that had statistically significant and/or strongest associations with each other: rMSSD (x), self-reported internalizing symptoms (y) and affective reactivity to activity-related stress (m). The results showed that affective reactivity to activity-related stress significantly mediated the association between rMSSD and internalizing symptoms in the whole sample (*b *= − 7.42, 95% CI [− 15.11, − 1.8], *p* = 0.009). Detailed results of the mediation analysis are presented in Table 4.

**Table 4:** Results of the bootstrap mediation analysis.

Effect of X on M (B)	Effect of M on Y (B)	Direct effect (B)	Indirect effect (B, 95% CI)	Total effect (B)
− 0.09*	75.93**	− 8.3	− 7.42 (− 15.11, − 1.8)**	− 15.72*

X = rMSSD (square root of the mean squared differences of successive R–R intervals).

M = Affective reactivity to activity-related stress.

Y = Self-reported internalizing symptoms.

*p < 0.05; **p < 0.01.

### Supplementary analyses: within-group investigation of associations

Given that group differences were observed in mental health symptoms (Table 1) as well as in daily affective dynamics, *post-hoc* analyses were conducted to investigate the associations between HRV, daily affective dynamics and mental health separately in each group. Mediation analysis was not conducted within groups due to small sizes of the subgroups.

When investigating the association between rMSSD and internalizing symptoms separately in the two groups, the results were not significant after multiple comparison correction in the autistic (*b *= − 0.02, 95% CI [− 0.03, − 0.005], *p* = 0.007, B–H threshold: *p* = 0.005) or in the non-autistic group (*b *= − 0.002, 95% CI [− 0.01,0.008], *p* = 0.713). However, in the autistic group, the model explained a substantial proportion of variance (R^2^ = 0.36, F(2,24) = 6.73, *p* = 0.005, adj. R^2^ = 0.31), whereas in the non-autistic group, the model explained a weak proportion of variance (R^2^ = 0.07, F(2,21) = 0.74, *p* = 0.489, adj. R^2^ = − 0.02) (see supplementary Fig. 1). Similarly, no other significant associations between HRV and mental health symptoms were observed in any group (data not shown). We also examined whether co-occuring psychiatric conditions or medication were linked with HRV in the autistic group, but no significant association was observed after multiple comparison correction (see supplementary material).

Concerning daily affective dynamics, the autistic group showed higher instability in negative affects (NA) (*b* = 0.35, 95% CI [0.09,0.60], *t* = 2.70, *p* = 0.01), but not in positive affects (PA) (*b* = 0.03, 95% CI [− 0.33,0.40], *t* = 0.17, *p* = 0.867) compared to the non-autistic group. However, when adding the mean level of NA as a covariate in the model, the group difference in NA was no longer significant (*b* = 0.09, 95% CI [− 0.10,0.53], *t* = 1.36, *p* = 0.530). Regarding affective reactivity to stress, the stress × group interaction was significant for event stress (*b* = 0.08, 95% CI [0.007,0.15], *p* = 0.032), indicating that autistic participants showed increased reactivity to event-related stress compared to the non-autistic group. In contrast, affective reactivity to activity-related (*b* = 0.02, 95% CI [− 0.07, 0.11], *p* = 0.673) or social stress (*b* = 0.05, 95% CI [− 0.12, 0.22], *p* = 0.586) did not differ between the groups.

Within-group analyses showed that, regarding the association between activity-related stress and NA, the moderating effect of rMSSD (*b *= − 0.26, 95% CI [− 0.48, − 0.04], *p* = 0.021) and HF (*b *= − 0.18, 95% CI [− 0.30, − 0.06], *p* = 0.004) was significant only in the autistic group but not in the non-autistic group (rMSSD: *b *= − 0.05, 95% CI [− 0.31,0.21], *p* = 0.705; HF: *b* = 0.001, 95% CI [− 0.14,0.14], *p* = 0.984). In contrast, the association between HRV and NA instability was significant only in non-autistic individuals (rMSSD: *b *= − 0.39, 95% CI [− 0.52, − 0.27], *t *= − 6.57, *p* < 0.001; HF: *b *= − 0.18, 95% CI [− 0.28, − 0.07], *t *= − 3.60, *p* = 0.002). The remaining effects were all non-significant (data not shown).

## Discussion

The current study aimed to investigate the interplay between resting HRV, daily affective dynamics and mental health symptoms in autistic and non-autistic adolescents and young adults. Our results showed that resting HRV did not differ between the two groups. HRV was not associated with mental health symptoms in analyses corrected for multiple comparisons, but it showed significant associations with daily affective dynamics. Furthermore, a mediation analysis showed that affective reactivity to stress mediated the association between lower HRV and more severe internalizing symptoms. Our findings suggest that increased affective reactivity to daily stressors may be a mechanism linking altered autonomic functioning to mental health difficulties, offering important insights for targeting clinical interventions in adolescents and young adults. Finally, differences emerged in the relationship between HRV and daily affective dynamics between the autistic and non-autistic groups, pointing toward the role of the environment in modulating daily affects in autistic adolescents.

### Resting HRV and mental health

No group differences in resting HRV were observed between non-autistic and autistic individuals, which contrasts with previous findings suggesting that altered autonomic functioning is an intrinsic characteristic of autism^21^. However, high inter-individual variability in HRV was observed in the autistic group, which is in line with a recent review^22^ arguing that previous group differences are driven by inter-individual factors, such as mental health symptoms. In the current study, we did not observe direct associations between HRV parameters and mental health symptoms, which contrasts with previous findings in the general population^6^ as well as in autistic samples^25,31^. However, we observed a stronger association between HRV and internalizing symptoms in the autistic group (with a regression model that explained a larger percentage of variance) than in the non-autistic group. This may be explained by the relatively low levels of internalizing symptoms in non-autistic participants and/or by other related mechanisms, such as the use of different adaptive emotion regulation strategies modulating the negative effects of stress.

On the other hand, the lack of significant results concerning the direct link between HRV and mental health symptoms in the autistic group may be due to several factors. First, it is possible that the relationship between HRV and mental health is not linear, which could have led to non-significant findings in our models. Indeed, a recent meta-analysis^5^ showed that most mental health conditions were associated with reduced HRV, but that higher HRV was observed in individuals with eating disorders, indicating an existence of an “ideal range”. Furthermore, the heterogeneity of our sample, particularly in terms of the age range, indicates that the duration and trajectory of mental health symptoms and/or conditions varied considerably between participants. Previous longitudinal studies have shown that HRV alterations can predict the onset of mental health difficulties^32^. Thus, an individual’s clinical trajectory may determine whether autonomic function is altered when the assessment is carried out. In addition, comorbidity is more a rule than an exception^33^, and likely influences the relationship between HRV and mental health difficulties. Previously, it has been shown that comorbid diagnoses may be associated with a greater reduction in HRV compared to a single diagnosis^34^. With this in mind, we investigated the influence of psychiatric comorbidities on HRV in the autistic group, but did not obtain any significant results, which may be due to the small size of the subsamples. The topic remains interesting for future studies.

It is also possible that psychotropic medication plays a role in the association between HRV and mental health symptoms, although it could not be systematically investigated in the current study. Previous studies have demonstrated the impact of psychotropic medication, such as antidepressants (which was the most common medication in our sample) on HRV, although there is no consensus on the topic. In some studies, the association between reduced HRV and mental health difficulties was driven by the effects of antidepressants^35,36^, whereas a meta-analysis showed reduced HRV also in medication naïve individuals^37^. The topic is complex because people with more severe symptoms are more likely to take medication, making it difficult to disentangle the effects on HRV. Future studies should consider the effect of psychotropic medication when investigating the association between autonomic functioning and mental health in autism.

### The role of daily affective dynamics

The results of the mediation analysis suggest that affective reactivity to stress may be a transdiagnostic mechanism linking altered autonomic functioning to mental health symptoms. It is however important to note that differences in daily affective dynamics and their association with HRV emerged between the two groups. Autistic youths showed greater NA instability than non-autistic participants, although the difference was no longer significant after controlling for the mean level of NA. Standard deviations also showed great within-person variability in NA/PA instability in the autistic group. This might indicate the existence of a subgroup of participants reporting increased affective instability, possibly increasing their vulnerability to develop mental health difficulties. Indeed, higher instability of NA was associated with more severe mental health symptoms, in line with earlier work^27^, suggesting that it could play a crucial role in the development of co-occuring mental health difficulties in autistic youth, and underlining the importance of working on emotion regulation strategies to reduce extreme emotional fluctuations. However, affective instability did not play a role in the association between HRV and mental health symptoms in autistic individuals. In contrast, the finding that NA instability was related to reduced HRV in the non-autistic sample is consistent with earlier research^16^ but raises questions about possible mechanisms that might explain the lack of such an association in the autistic group. One such might be alexithymia, i.e., difficulty to identify, differentiate and express one’s emotions, which is common in autism^38^ and likely influences the assessment and regulation of daily affects. In a recent EMA study, autistic adults showed more momentary experiences of alexithymia than non-autistic adults, and these experiences were associated with reduced control of one’s emotions^39^. To better understand the role of alexithymia and target interventions, it would be important to further investigate its impact on the modulation and self-evaluation of daily affects in autism.

Regarding affective reactivity to stress, autistic participants showed an increased affective reactivity to event-related stressors compared to non-autistic participants, in line with earlier results of heightened stress reactivity in autism^28,40^, which was linked with the severity of mental health symptoms. However, the nature of the daily stressors associated with increased affective reactivity does not appear to be constant across samples^28^. The EMA period could contribute to the different results between the samples: in the present study, autistic participants were mostly assessed during a period without structured activity, which likely influenced the nature and the number of daily stressors as well as the intensity of their affects compared to a school/working period. Stressors related to daily activities appeared to have a specific role in the current study, given the unique association between resting HRV and increased reactivity to activity-related stress, as well as the latter’s role in mediating the association between HRV and internalizing symptoms. It is possible that poor person-environment fit^41^ explains the importance of activity-related stressors. Due to some core characteristics of autism, such as sensibility to sensory stimuli and importance of routines, some daily-life environments might not be adapted to specific needs of individuals, increasing the negative impact of stress related to their daily activities. The hypothesis of poor person-environment fit could also support the important observation that the environment had an increased impact on the modulation of daily affects in autistic individuals, given that only affective reactivity to stress (and not affective instability) was associated with resting HRV and implicated in the interplay between autonomic control and mental health.

### Clinical perspectives

As a priority, our results highlight the importance on working on adaptative regulation of daily affects in order to prevent negative outcomes of stress. This appears to be important for autistic youths, in particular, as they showed increased affective reactivity to stressors. Specific techniques, such as dialectical behavior therapy, cognitive behavioral therapy or mindfulness-based techniques could be helpful in autistic individuals^42–44^. Furthermore, given the fact that environment appeared to play an important role in the modulation of daily affects in youths with autism, it seems essential to decrease environmental stressors by making environmental adjustments. These could include, among others, avoiding last-minute changes and taking into account the importance of routines, avoiding loud noises or bright lights, and using special equipment such as noise-canceling headphones.

### Limitations

The results should be considered in the light of certain limitations. The previous literature has shown that various factors can affect HRV^45^. Due to our study design, we could not standardize the time of the measurement or the last meal, as recommended for HRV data collection^46^. However, as the number of participants who had eaten (or consumed caffeine/tobacco) less than two hours prior to measurement was small, we decided to add these variables as covariates. The measurement time was also controlled for and did not influence HRV parameters. Moreover, other factors, such as participants’ physical condition, could influence resting HRV, but these were not investigated in the current study.

Considering the subjectivity of EMA assessments, the interpretation of questions could have differed between individuals. However, all the items were reviewed with participants during installation to minimize this issue. Moreover, the autistic youths participated in the EMA protocol mainly during a period without structured activity, unlike the non-autistic participants, which may have influenced the results. Finally, it should be noted that the autistic sample was verbally fluent and had mostly (above) average intellectual functioning, so the results cannot be generalized to all autistic youths.

## Conclusions

The current study showed no group differences in resting HRV between autistic and non-autistic adolescents and young adults, contrasting some previous findings and suggesting that autonomic function in autism is not altered per se, but that there is high inter-individual variability within individuals. We found no direct associations between HRV and mental health difficulties, and further studies are needed to better understand the role of HRV in mental health in autism. Importantly, our results suggest that increased affective reactivity to daily stressors may be a transdiagnostic mechanism linking altered autonomic function to mental health difficulties. This underscores the importance of working on adaptive emotion regulation to prevent negative effects of stress in adolescence and young adulthood, particularly in autistic individuals who showed heightened affective reactivity to stress. Finally, our results show that the environment had an increased importance in modulating daily affects in autistic youths, which emphasizes the need to reduce environmental stressors.

## Methods

### Sample

The sample consisted of 31 verbally fluent participants with a confirmed diagnosis of ASD (based on DSM-5 criteria) and 35 non-autistic participants, aged 12–30 years. Autistic participants were assessed with the Autism Diagnostic Observation Schedule, second version (ADOS-2)^47^, and with their caregivers, the Autism Diagnostic Interview-Revised (ADI-R)^48^ and/or the Social Communication Questionnaire (SCQ)^49^ was completed. For non-autistic participants, the exclusion criteria included premature birth, presence of neurological issues or neurodevelopmental/psychiatric disorders (except for a past psychiatric disorder in complete remission), and having a first-degree relative with neurodevelopmental disorder (except for a de novo neurogenetic condition). Intellectual functioning was assessed with Weschler Intelligence Scale for Children/Adults^50,51^, with none of the participants having an IQ score within the intellectual disability range. All participants and caregivers gave their written consent. Participants received a financial compensation of 100 Swiss francs for participating in a larger study including additional measures. The study was approved by the Cantonal Research Ethics Committee of Geneva (CCER) (2018 − 01117). Further information on data collection and recruitment is provided in supplementary material.

## Measures

### Mental health symptoms

Mental health symptoms were measured through self-reported and caregiver-reported questionnaires: for children and adolescents, the Youth Self Report (YSR) and the Child Behavior Checklist (CBCL)^52^ and for adults, the Adult Self Report (ASR) and the Adult Behavior Checklist (ABCL)^53^. The age-normalized T-scores of internalizing and externalizing symptoms were used in the analyses.

Social anxiety was assessed using the Social Interaction Anxiety Scale (SIAS)^54^, a self-reported questionnaire that measures participants’ fear of social interactions. Participants responded using a 5-point Likert scale (“not at all” to “extremely characteristic/true of me”), with higher scores corresponding to more severe social anxiety.

With autistic participants, a comprehensive clinical assessment was conducted to examine the presence of co-occurring conditions (see supplementary material).

### Resting HRV

HRV was measured at rest in the laboratory once for each participant using a mobile electrocardiogram (ECG) recorder Bittium^®^ Faros 180 (Bittium Corporation) attached to a chest belt. The sampling rate was 250 Hz. The measurement was conducted in the laboratory because HRV is extremely sensitive to various factors, including physical activity, respiratory rate and posture^55^, and we wanted to ensure that conditions were similar for each participant. Participants were instructed to sit with their knees at a 90-degree angle, feet on the floor and hands on thighs for 10 min while watching a neutral documentary film about trees. A neutral documentary film was chosen because it has previously been used to obtain resting HRV measurements [e.g., 56, 57]. The use of a neural film ensures similar conditions for all participants and may prevent arousing thoughts and feelings (negative or positive) that could influence HRV measurements, which may be more likely to occur during a “task-free state” when participants’ minds wander. To ensure acclimatization to the recording environment^46^, an ECG was recorded for the entire 10 min, but the first five minutes of the recording were not included in the analyses. Therefore, the length of the analyzed ECG record was five minutes. The measurements were taken between 9.00 and 17.00, ensuring that participants had not eaten, drunk caffeine or smoked tobacco for at least two hours prior to the measurement. If this was not possible (in some rare cases), recent eating (*n* = 7), drinking caffeine (*n* = 3) and smoking (*n* = 1) was recorded and controlled in the analyses to avoid the loss of data. Moreover, intense physical activity on the previous/same day (*n* = 4) as well as alcohol consumption in the 24 h preceding the measurement (*n* = 5) were noted and controlled. The ECG data were first inspected using the Cardiscope™ Analytics software. Due to measurement problems, 4 autistic and 11 non-autistic participants were excluded.

For the valid records, R–R intervals were exported to R^58^ for the data processing using the *RHRV* package^59^. A filter using adaptive thresholding was used to automatically remove artifacts, i.e., R–R intervals that exceeded the cumulative mean threshold and beats whose values were not within acceptable physiological range (*n* = 121). Moreover, data were visually inspected, and 46 artifacts were manually removed. Resting HRV was derived from R–R intervals for 60-s windows that were averaged over the 5-minute record using two different parameters; a time-domain method included the square root of the mean squared differences of successive R–R intervals (rMSSD) and a frequency-domain method included a high frequency (HF:0.15–0.4 Hz) power. Both parameters reflect parasympathetic activity^3^ and are strongly correlated^60^. However, rMSSD has shown to be less affected by the respiration rate^61^.

### Ecological momentary assessment (EMA)

A smartphone-based EMA was used to evaluate participants’ affects and perceived stress in their daily lives. During their visit to the laboratory, the RealLife Exp application (associated to the Lifedatacorp platform) was installed on the smartphone of each participant. During the installation, participants received detailed information about the EMA protocol and completed a test questionnaire with the examiner, being informed that the response period would begin the following day. A semi-random signal-contingent sampling scheme was used with eight notifications per day for six consecutive days between 07:30 and 22:00 (with a window of at least 30 min between two notifications), resulting in a maximum of 48 beeps per person. The period during which each participant took part in the EMA assessment was recorded (either: structured activity, i.e., school/work, or no structured activity, i.e., holidays/ without professional activity). Further details of the EMA protocol and the psychometric properties of the measures are provided in the supplementary material.

At each beep, momentary positive (PA) and negative (NA) affects and perceived stress were measured on a scale from 1 (not at all) to 7 (extremely). Measures are detailed in Table 5. Measures of affect and perceived stress were used to calculate daily affective dynamics as follows.

**Table 5:** Ecological momentary assessment items and measures.

Positive affects (PA)	Positive affects were assessed using the mean score of the following items: I feel relaxed, I feel content/cheerful, I feel excited, I have confidence in myself
Negative affects (NA)	Negative affects were assessed using the mean score of the following items: *I feel alone*, *I feel anxious*, *I feel irritated/angry*, *I feel sad*
Event-related stress	To assess event-related stress, participants were asked to think about the most important event that happened since the last beep and evaluate it with the following item: *“This event was enjoyable”* (reversed score)
Activity-related stress	To assess activity-related stress, participants evaluated their current activity with the following items: *This activity is difficult*, and *I enjoy doing this activity* (reversed score)
Social stress	Social stress was assessed during the beeps when participants reported being in the company of others using the following items: *I would prefer to be alone*, *This company is pleasant* (reversed score), *I feel judged by this/these person(s)*, *I feel nervous in the presence of this/these person(s)*

*Affective reactivity to stress* To estimate participants’ individual level of affective reactivity to stress, a multilevel regression model with random intercepts and random slopes was performed, using NA as the dependent variable and stress as the independent variable (separate models for event, activity and social stress). The slopes of these regression models were extracted for each participant, presenting the individual level of affective reactivity to stress (i.e., association between stress and negative affect in each context of stress).

*Affective instability* Participants’ individual level of affective instability was assessed by calculating the mean square successive difference (MSSD) separately for positive and negative affects for each participant^62^. MSSD was used, as it considers the variability and the temporal dependency of affects^63^. Difference scores between evening and morning beeps were excluded to remove changes in affect between different days^16^. In case of missing notifications, a lag of up to two beeps was allowed.

### Statistical analyses

The analyses were conducted using R version 4.4.2^58^. HRV parameters (rMSSD and HF) were log_10_ transformed to correct for skewness. Prior to the transformation, two outliers (> 3SD) were observed, while no outliers occurred after log_10_ transformation. In order to avoid multicollinearity, the two HRV parameters that were strongly correlated were never included in the same regression models, but separate analyses were conducted using either rMSSD or HF in the models. Multiple linear regression was used to test the effect of multiple covariates (age, sex, BMI, IQ, regular smoking, measurement time, alcohol consumption, physical training, recent eating, recent caffeine drinking and recent smoking) on HRV. As the only significant variable influencing HRV was recent eating, it was retained in the models. In analyses including EMA variables, EMA period was added as a covariate, but as the effect was not significant, it was not included in the final models. Time-invariant (one value per person) variables (covariates, scores of mental health symptoms) were grand-mean centered (i.e., the mean of the entire dataset for the specific variable was subtracted from each individual’s score to create a new, centered variable where the values were deviations from the grand mean). For multiple linear regression models, multiple comparisons were corrected using the Benjamini–Hochberg (B–H) correction^64^. The magnitude of adjusted R^2^ values were interpreted using the thresholds of Cohen^65^.

First, due to non-normal distribution of all variables, group differences in demographic characteristics and questionnaires were examined using Mann-Whitney U and chi square tests. Group differences in HRV parameters were tested using multiple linear regression, whereas robust regression was used for affective instability. Due to the two-level structure of EMA data (repeated measures nested withing individuals), multilevel regression models were used to investigate group differences in affective reactivity to stress.

Second, multiple linear regression models were used to examine associations between HRV and mental health symptoms. Several models were run with different outcome variables (internalizing/externalizing symptoms/social anxiety) and HRV as a predictor.

Third, regarding affective instability, robust regression (due to few outliers and non-normal distribution of residuals) was conducted using MSSD of PA/NA as dependent variable and HRV as independent variable. Individual mean level of PA/NA was added as a covariate, as recommended^62^. Then, to investigate whether HRV moderated the association between different types of daily-life stress and NA, several multilevel regression models with random intercepts and random slopes were run (separately for social stress, activity-related stress and event-related stress). Daily stress, HRV and their interaction were used as independent variables and NA as the dependent variable.

Finally, a mediation analysis was conducted using a robust bootstrap test for mediation analysis ROBMED^66^ due to nonnormal distribution. Bootstrapping does not require the assumption of normal distribution and it reduces the Type I error rate^67^.

The current study was pre-registered during data collection (10.17605/OSF.IO/KYTCX). Some deviations from the preregistration were made and are detailed in the supplementary material.

## Supplementary Information

Below is the link to the electronic supplementary material.

Supplementary Material 1

## Data Availability

The dataset used in the current study will be available in the YARETA data repository.
