# Supplementary material for: Interplay between resting heart rate variability, daily affective dynamics and mental health difficulties in autistic youths
Source: Sci Rep. 2026 Jan 6;16:4495. doi: 10.1038/s41598-025-34614-y (PMC12864953; doi:10.1038/s41598-025-34614-y)
Supplement: Supplementary file 1 — Supplementary Material 1 [file 41598_2025_34614_MOESM1_ESM.docx]

***Interplay between resting heart rate variability, daily affective dynamics and mental health difficulties in autistic youths***

*Laura Ilen^a*^, Julie Husmann^a^, Clémence Feller^a^, Maude Schneider^a^*

*^a^Clinical Psychology Unit for Intellectual and Developmental Disabilities, Faculty of Psychology and Educational Sciences, University of Geneva, Geneva, Switzerland*

**Supplementary material**

**Methods**

**Data collection and sample**

Data were collected between August 2022 and September 2024. Autistic participants were recruited through clinical centers in Geneva and France, through a network of medical professionals and through announcements to family associations in Switzerland and France. The non-autistic group was recruited through an ongoing longitudinal cohort involving typically developing participants and youths with a neurogenetic condition (see Sandini et al., 2020). Of note, part of the non-autistic sample (*n*=15) overlapped with that of our earlier study using the EMA (Ilen et al., 2023). However, as participants were evaluated through longitudinal cohort, they were assessed at a different time point. There was no overlap regarding the autistic sample.

**Assessment of co-occurring psychiatric conditions**

A comprehensive clinical assessment was conducted with autistic participants to examine the presence of DSM-5 diagnoses. The Schedule for Affective Disorders and Schizophrenia for School-Age Children – Present and Lifetime version DSM-5 (K-SADS-PL-5) (Kaufman et al., 1997) was used with participants below 18 years old, whereas the Structured Clinical Interview for Axis I DSM-IV/DSM-5 (SCID) (Spitzer et al., 1992) was used with adult participants.

**Deviations from the preregistration**

The current study was pre-registered during data collection (<https://doi.org/10.17605/OSF.IO/KYTCX>). Some deviations from the preregistration were made. The main change concerned the population of the study. Initially, we had also planned to include a group of young people with a neurogenetic condition, 22q11.2 deletion syndrome (22q11DS), but after inspection of the data, their HRV profile was very different from that of the two other groups, which would have required separate analyses. The difference could be linked to the wide range of somatic manifestations, in particular congenital heart diseases, as well as high rates of psychiatric symptoms associated with the syndrome (Óskarsdóttir et al., 2023) and are worthy of more in-depth investigation. Therefore, we decided to exclude the 22q11DS group from the current manuscript and investigate their HRV profile in a forthcoming publication. In addition, some deviations concerning the analyses were conducted. First, considering the impact of circadian rhythm on HRV (Massin et al., 2000), time of the HRV measure was added as a covariate in the analyses. However, as it did not have a significant effect, it was finally not retained in the models. Moreover, in analyses on affective instability, we used MSSD and robust regression instead of time series of SSD and multilevel model, as initially planned. This was done as NA and PA instability variables included a very large number of zero or very low values, making the distribution highly skewed and problematic for analysis. Finally, the mediation analysis was conducted using a robust bootstrap test for mediation instead of a Sobel test, as it is more suitable for nonnormal data (Alfons et al., 2022).

**Details on the EMA protocol**

For the EMA assessment, the RealLife Exp application (associated to the Lifedatacorp platform) was installed on the smartphone of each participant. During the installation, participants were briefed extensively about the EMA protocol and completed a test questionnaire with the examiner. A semi-random signal-contingent sampling scheme was used with eight notifications per day for six consecutive days between 07:30 am and 22:30 pm, resulting in a maximum of 48 beeps per person. A window of at least 30 minutes was scheduled between two consecutive notifications. Participants were instructed to respond to a short questionnaire on the application within 15 minutes after receiving a notification. The notifications completed in more than 15 minutes were excluded (*n*=15), to ensure that participants' responses reflected the present moment. Moreover, all the notifications completed in less than one minute were examined individually, but no clear pattern of careless responding (i.e., the same response to each item) was observed. In line with earlier studies (Myin-Germeys et al., 2001), participants needed to complete at least one-third of the beeps (*n*=16) to be included in the analyses, which was the case for all the participants.

**Psychometric properties of EMA measures**

To investigate the factor structure of affect items, within-person centered data were entered in a factor analysis (principal component analysis with oblimin rotation), which revealed a two-factor structure of affect items. Positive affects (PA) were assessed using the mean score of the following items: *I feel relaxed, I feel content/cheerful, I feel excited, I have confidence in myself* (respective loadings were 0.42, 0.70, 0.84 and 0.57; Cronbach’s α = 0.64). Negative affects (NA) were assessed using the mean score of the following items: *I feel alone*, *I feel anxious*, *I feel irritated/angry*, *I feel sad* (respective loadings were 0.76, 0.56, 0.51, 0.76; Cronbach’s α = 0.63).

Regarding stress variables, the Cronbach’s α was 0.51 for activity-related stress, which consisted of two items: *This activity is difficult*, and *I enjoy doing this activity* (reverse-scored). For social stress, the Cronbach’s α was 0.75 (items: *I would prefer to be alone*, *This company is pleasant* (reversed score), *I feel judged by this/these person(s)*, *I feel nervous in the presence of this/these person(s)*).

**Results**

**Effect of psychiatric diagnosis and psychotropic medication on HRV in the autistic sample**

The effect of current mood and/or anxiety disorders on HRV was investigated in autistic participants. Of note, none of the participants had a mood disorder without an associated anxiety disorder. In individuals with both disorders (*n*=4), no significant differences compared to group without clinical diagnoses were observed in rMSSD (*b*=-0.33, 95% CI [-0.61, -0.05], *t*(24)=-2.42, *p*=0.023) or HF (*b*=-0.58, 95% CI [-1.07, -0.09], *t*(24)=-2.43, *p*=0.023) was observed after multiple comparison correction. Similarly, there was no significant difference in terms of rMSSD (*b*=-0.12, 95% CI [-0.26,0.02], *t*(48)=-1.66, *p*=0.103) or HF (*b*=-0.14, 95% CI [-0.39,0.11], *t*(48)=-1.14, *p*=0.262) in individuals with an anxiety disorder without associated mood disorder compared to individuals without psychiatric diagnoses (*n*=16).

Finally, the effect of current psychotropic medication on the HRV-mental health association in autistic participants was investigated by adding a dichotomic variable (yes/no) for medication use as a covariate in the models. Due to the considerable overlap between the use of antidepressants, anxiolytics and antipsychotics (only one individual taking anxiolytic or antipsychotic medication was not also taking an antidepressant), it was not possible to investigate the effect of each medication separately, and these three medications were combined in the analyses. When covarying for the use of these medications, HRV was not associated with the diagnosis of mood and anxiety disorder. Furthermore, covarying for the use of psychostimulants did not significantly change the results.

**Supplementary Table 1.** Associations between HRV parameters and mental health in the autistic group, after controlling for the use of current psychotropic medication

|  | | ***β* (95% CI)** | ***t*** | ***p*** |
| --- | --- | --- | --- | --- |
| *Covariate: antidepressants, anxiolytics and/or antipsychotics* | |  |  |  |
| **Mood and anxiety disorder** | |  |  |  |
| rMSSD | -0.25 (-0.57, 0.06) | -1.65 | 0.113 |  |
| HF | -0.47 (-1.03, 0.09) | -1.75 | 0.094 |  |
| *Covariate: psychostimulants* | |  |  |  |
| **Mood and anxiety disorder** | |  |  |  |
| rMSSD | -0.31 (-0.60, -0.02) | -2.20 | 0.038 |  |
| HF | -0.55 (-1.06, -0.04) | -2.22 | 0.036 |  |

Note: p-values did not survive multiple comparison correction (B-H)

HF: high-frequency power, rMMSD: square root of the mean squared differences of successive R-R intervals


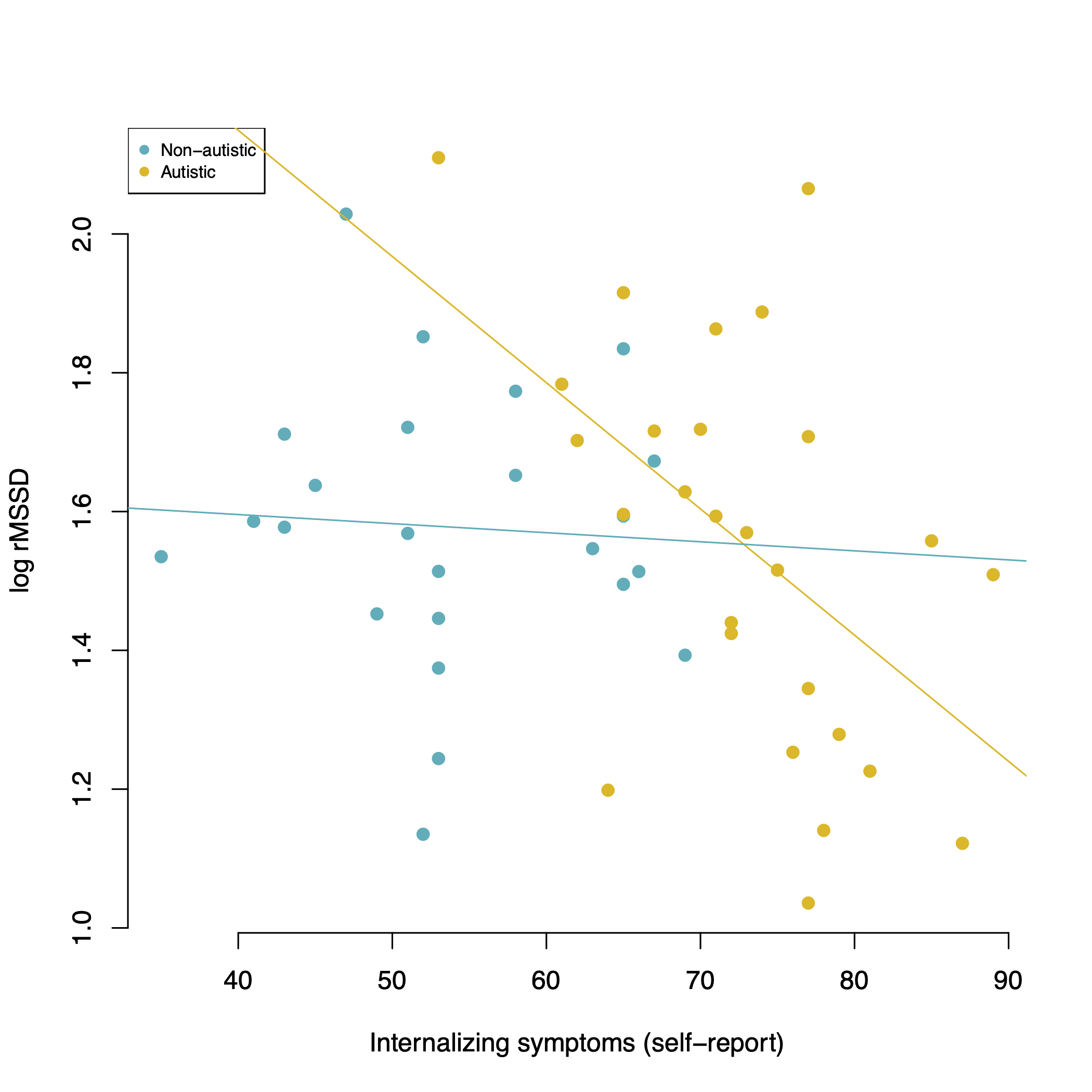


**Supplementary Figure 1.** Log_10_-transformed HRV (rMSSD) values plotted against the scores of self-reported internalizing symptoms in each group

**Supplementary Table 2.** Results of linear regression models on associations between daily affective dynamics and mental health symptoms in the entire sample

|  | | Social anxiety | Internalizing (self) | Internalizing (parent) | Externalizing (self) | Externalizing (parent) |
| --- | --- | --- | --- | --- | --- | --- |
| NA instability | **t(49)=2.89, p=0.006*** | **t(49)=4.61, p<0.001*** | **t(49)=3.57, p<0.001*** | **t(49)=4.66, p<0.001*** | t(49)=1.99, p=0.052 |  |
| PA instability | t(49)=1.46, p=0.152 | t(49)=1.49, p=0.143 | t(49)=1.05, p=0.298 | t(49)=2.05, p=0.045 | t(49)=1.18, p=0.245 |  |
| Event stress reactivity | **t(49)=3.31, p=0.002*** | **t(49)=4.92, p<0.001*** | **t(49)=3.90, p<0.001*** | **t(49)=3.66, p<0.001*** | t(49)=2.17, p=0.035 |  |
| Activity stress reactivity | t(49)=2.69, p=0.010 | **t(49)=3.24, p=0.002*** | t(49)=2.07, p=0.044 | t(49)=2.36, p=0.022 | t(49)=0.49, p=0.624 |  |
| Social stress reactivity | t(49)=1.82, p=0.075 | **t(49)=3.33, p=0.002*** | **t(49)=2.86, p=0.006*** | **t(49)=2.79, p=0.007*** | t(49)=1.18, p=0.244 |  |

******p-values surviving the Benjamini-Hochberg correction for multiple comparisons*

Alfons, A., Ateş, N. Y., & Groenen, P. J. (2022). A robust bootstrap test for mediation analysis. *Organizational Research Methods*, *25*(3), 591-617.

Ilen, L., Feller, C., & Schneider, M. (2023). Cognitive emotion regulation difficulties increase affective reactivity to daily-life stress in autistic adolescents and young adults. *Autism*, 13623613231204829. <https://doi.org/10.1177/13623613231204829>

Kaufman, J., Birmaher, B., Brent, D., Rao, U., Flynn, C., Moreci, P., Williamson, D., & Ryan, N. (1997). Schedule for Affective Disorders and Schizophrenia for School-Age Children-Present and Lifetime Version (K-SADS-PL): initial reliability and validity data. *Journal of the American Academy of Child and Adolescent Psychiatry*, *36*(7), 980-988. <https://doi.org/10.1097/00004583-199707000-00021>

Massin, M. M., Maeyns, K., Withofs, N., Ravet, F., & Gérard, P. (2000). Circadian rhythm of heart rate and heart rate variability. *Arch Dis Child*, *83*(2), 179-182. <https://doi.org/10.1136/adc.83.2.179>

Myin-Germeys, I., van Os, J., Schwartz, J. E., Stone, A. A., & Delespaul, P. A. (2001). Emotional reactivity to daily life stress in psychosis. *Archives of general psychiatry*, *58*(12), 1137-1144. <https://doi.org/10.1001/archpsyc.58.12.1137>

Óskarsdóttir, S., Boot, E., Crowley, T. B., Loo, J. C. Y., Arganbright, J. M., Armando, M., Baylis, A. L., Breetvelt, E. J., Castelein, R. M., Chadehumbe, M., Cielo, C. M., de Reuver, S., Eliez, S., Fiksinski, A. M., Forbes, B. J., Gallagher, E., Hopkins, S. E., Jackson, O. A., Levitz-Katz, L., . . . McDonald-McGinn, D. M. (2023). Updated clinical practice recommendations for managing children with 22q11.2 deletion syndrome. *Genet Med*, *25*(3), 100338. <https://doi.org/10.1016/j.gim.2022.11.006>

Sandini, C., Chambaz, M., Schneider, M., Armando, M., Zöller, D., Schaer, M., Sandi, C., Van De Ville, D., & Eliez, S. (2020). Pituitary dysmaturation affects psychopathology and neurodevelopment in 22q11. 2 Deletion Syndrome. *Psychoneuroendocrinology*, *113*, 104540. <https://doi.org/10.1016/j.psyneuen.2019.104540>

Spitzer, R. L., Williams, J. B., Gibbon, M., & First, M. B. (1992). The Structured Clinical Interview for DSM-III-R (SCID). I: History, rationale, and description. *Archives of general psychiatry*, *49*(8), 624-629. <https://doi.org/10.1001/archpsyc.1992.01820080032005>
